# Exploring Anti-Bacterial Compounds against Intracellular *Legionella*

**DOI:** 10.1371/journal.pone.0074813

**Published:** 2013-09-13

**Authors:** Christopher F. Harrison, Sébastien Kicka, Valentin Trofimov, Kathrin Berschl, Hajer Ouertatani-Sakouhi, Nikolaus Ackermann, Christian Hedberg, Pierre Cosson, Thierry Soldati, Hubert Hilbi

**Affiliations:** 1 Max von Pettenkofer Institute, Ludwig-Maximilians University, Munich, Germany; 2 Department of Biochemistry, University of Geneva, Geneva, Switzerland; 3 Faculty of Medicine, University of Geneva, Geneva, Switzerland; 4 Max Planck Institute of Molecular Physiology, Dortmund, Germany; University of Lausanne, Switzerland

## Abstract

*Legionella pneumophila* is a ubiquitous fresh-water bacterium which reproduces within its erstwhile predators, environmental amoeba, by subverting the normal pathway of phagocytosis and degradation. The molecular mechanisms which confer resistance to amoeba are apparently conserved and also allow replication within macrophages. Thus, *L. pneumophila* can act as an ‘accidental’ human pathogen and cause a severe pneumonia known as Legionnaires’ disease. The intracellular localisation of *L. pneumophila* protects it from some antibiotics, and this fact must be taken into account to develop new anti-bacterial compounds. In addition, the intracellular lifestyle of *L. pneumophila* may render the bacteria susceptible to compounds diminishing bacterial virulence and decreasing intracellular survival and replication of this pathogen. The development of a single infection cycle intracellular replication assay using GFP-producing *L. pneumophila* and *Acanthamoeba* *castellanii* amoeba is reported here. This fluorescence-based assay allows for continuous monitoring of intracellular replication rates, revealing the effect of bacterial gene deletions or drug treatment. To examine how perturbations of the host cell affect *L. pneumophila* replication, several known host-targeting compounds were tested, including modulators of cytoskeletal dynamics, vesicle scission and Ras GTPase localisation. Our results reveal a hitherto unrealized potential antibiotic property of the β-lactone-based Ras depalmitoylation inhibitor palmostatin M, but not the closely related inhibitor palmostatin B. Further characterisation indicated that this compound caused specific growth inhibition of *Legionella* and *Mycobacterium* species, suggesting that it may act on a common bacterial target.

## Introduction

*Legionella pneumophila* is a ubiquitous environmental bacterium found in a wide range of fresh-water sources [1,2]. Bacteria within these environments suffer continuous predation from protozoa and as such have evolved a range of mechanisms to survive [3]. *L. pneumophila* produces a number of effector proteins which are injected into the host cell upon uptake. These effector proteins subvert the phagosome maturation process, instead promoting the formation of a biochemically distinct and replication-permissive “ *Legionella* -containing vacuole” (LCV) [4,5,6]. The molecular processes by which this occurs are aimed at evolutionarily conserved molecular targets and are also utilized following uptake by human alveolar macrophages, thereby subverting a crucial cellular component of the innate immune system and allowing *Legionella* to act as an ‘accidental’ pathogen [7]. Through this process the inhalation of *L. pneumophila* can lead to the often fatal pneumonia known as Legionnaires’ disease.

The intracellular localization of *L. pneumophila* provides a challenge for targeting and eliminating the bacteria both in the environment and in patients. *L. pneumophila* within water systems such as air cooling ducts are commonly found residing within free-living amoeba, where they are protected from chemical decontamination [8,9]. Similarly, *L. pneumophila* within macrophages are more resistant to antibiotics, partly due to poor intracellular penetration of the compounds [10]. In addition, bacteria released from phagocytic cells are much more resistant to antibiotic treatment than their counterparts grown in broth alone [11].

Legionnaires’ disease is most commonly treated with combinations of macrolide and quinolone antibiotics, however despite treatment the mortality rate of infected individuals ranges from 2 to 5% [12]. There is thus a need to develop alternative, more effective methods of treating *Legionella* infections. Beyond its importance as a human pathogen, *L. pneumophila* subverts similar host processes as other vacuolar bacteria [13], and thus may allow development of generic compounds against intracellular pathogens.

High throughput screening (HTS) was developed as a method to analyse many thousands of ‘drug-like’ compounds for effects on proteins or species of interest. However, the role of HTS in antibacterial development remains contentious, as large scale efforts to develop new broad-spectrum antibiotics met with very limited success [14]. From this the concept of anti-virulence has emerged as a promising anti-bacterial strategy. Anti-virulence compounds target bacterial processes that are only used when the pathogen resides within a host and are not *per se* required for extracellular survival and replication [15,16,17,18]. Essentially, anti-virulence compounds target bacterial pathogenicity, rather than viability. Another class of anti-infective compounds boosts host cell defence mechanisms, thereby restraining intracellular growth of pathogens. The identification of anti-virulence or defence-enhancing compounds requires an easy and robust assay for intracellular growth. Whereas numerous methods have been developed to examine the properties of intracellular *L. pneumophila* [19,20,21], the majority of these are not suitable for screening large numbers of compounds. The disadvantages of published assays include a high degree of complexity, lengthy setup and assay times, and often the requirement for charcoal-based media which can absorb the compounds being tested.

This report documents the development of a robust, non-invasive assay in 96-well format, which measures the effect of compounds on the replication of fluorescent *L. pneumophila* within the model host amoeba *A* *. castellanii* . The conditions of this assay can be modified to examine replication within other host cells such as the social amoeba *Dictyostelium discoideum* or murine RAW 264.7 macrophages. The initial analysis revealed an unexpected antibacterial property of the β-lactone-based thioesterase inhibitor palmostatin M. This compound specifically inhibits the growth of members of the genera *Legionella* and *Mycobacterium* , suggesting a common target or mode of action.

## Results

### Single infection cycle intracellular replication of GFP-producing *L. pneumophila*

**Figure 1:**
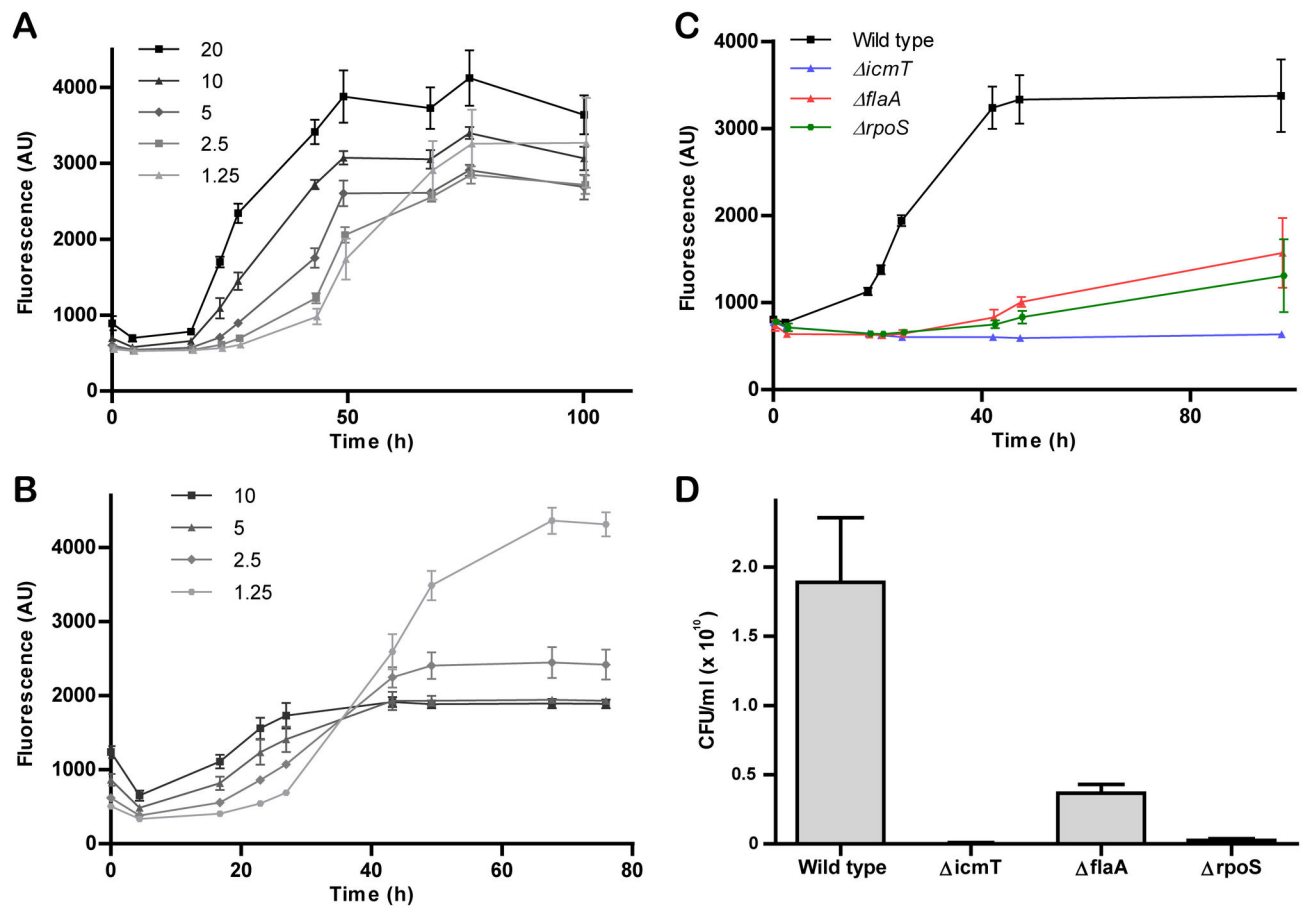
Single infection cycle intracellular replication of GFP-producing *L. pneumophila*. **A**-**B**. The phagocytic host cells (**A**) *A* *. castellanii* , or (**B**) RAW 264.7 macrophages were infected in 96-well plates with GFP-producing *L. pneumophila* at various MOIs, and the progress of intracellular growth was followed by fluorescence measurement using a microtiter plate reader. **C**. *A* *. castellanii* was infected with wild-type *L. pneumophila* or a Δ*icmT*, Δ*flaA* or Δ*rpoS* mutant strain (MOI 20), and the progress of replication was followed by fluorescence measurement. **D**. Representative infected wells from (**C**) were lysed after 48 h and the colony forming units (CFU) determined by plating on CYE agar. The graphs are representative of at least 3 independent experiments and indicate the average of three separate infections; error bars indicate 95% confidence intervals.

Testing anti-virulence, defence-boosting or antibiotic compounds requires a robust and complementary combination of infectious bacteria and replication-permissive host cells. *L. pneumophila* constitutively expressing GFP was utilised to allow non-invasive monitoring of intracellular replication. Protocols were developed for the infection of the environmental amoebae *A* *. castellanii* and *D. discoideum*, as well as the murine RAW 264.7 macrophage line. The ubiquitous amoeba *A* *. castellanii* , a common environmental host of *L. pneumophila* [1], was determined to be the most appropriate and robust host, with rapid, approximately 5-fold bacterial replication observed within 50 h (Figure 1A). The choice of LoFlo amoeba culture medium prevented *L. pneumophila* growth outside the cells while minimising auto-fluorescence. Multiplicities of infection (MOIs) above one ensured that the majority of the amoebae were infected at the beginning, and thus only a single round of intracellular replication was observed. Bacterial replication stopped after 50 to 100 h, depending on the MOI, suggesting an upper limit for the capacity of the amoeba to support the replication of intracellular bacteria (Figure 1A).

Intracellular replication was also examined within the commonly used, genetically tractable amoeba *D. discoideum* (Figure S1). The assays were performed at 25°C, as *D. discoideum* is unable to survive at higher temperatures for a prolonged time [22], which in turn led to a significantly slower (4-5 fold) replication over 150 h compared to growth in *A* *. castellanii* . For these reasons *D. discoideum* was deemed less suitable than *A* *. castellanii* for high- or medium-throughput compound screens. Further adaptation of the assay allowed the measurement of intracellular replication within murine RAW 264.7 macrophages (Figure 1B). Replication was less effective than in amoebae; with only a 2-3 fold increase observed after 50 h. Surprisingly, infection at low MOIs (2.5 or lower) resulted in an approximately 3-fold higher final bacterial load than that seen at higher MOIs. Owing to its robustness, speed and efficiency *A* *. castellanii* was utilised for initial compound testing, followed by validation using the more pathologically relevant macrophage cell line.

Having established the conditions for measuring intracellular growth in *A* *. castellanii* , the properties of known *L. pneumophila* deletion mutants were next examined (Figure 1C). Wild-type *L. pneumophila* replicated efficiently and reached a plateau phase approximately 50 h post-infection. In stark contrast, deletion of the Icm/Dot subunit IcmT, which impairs the T4SS and leaves the mutant unable to form a replication-permissive vacuole [23], led to the destruction of bacteria within 6 h. Deletion of the *L. pneumophila* alternative sigma factor RpoS (σ^S^/σ^38^) or the flagellum subunit FlaA led to significantly reduced intracellular replication, in line with previous publications [24,25]. The reduced replication observed via fluorescence was verified by counting the colony forming units (CFU) present after 2 days (Figure 1D, Figure S1). Thus, the single round fluorescence assay developed here correlates well with a classical CFU counting assay, while being faster and allowing higher throughput.

### Inhibition of intracellular replication by low molecular weight compounds

**Figure 2:**
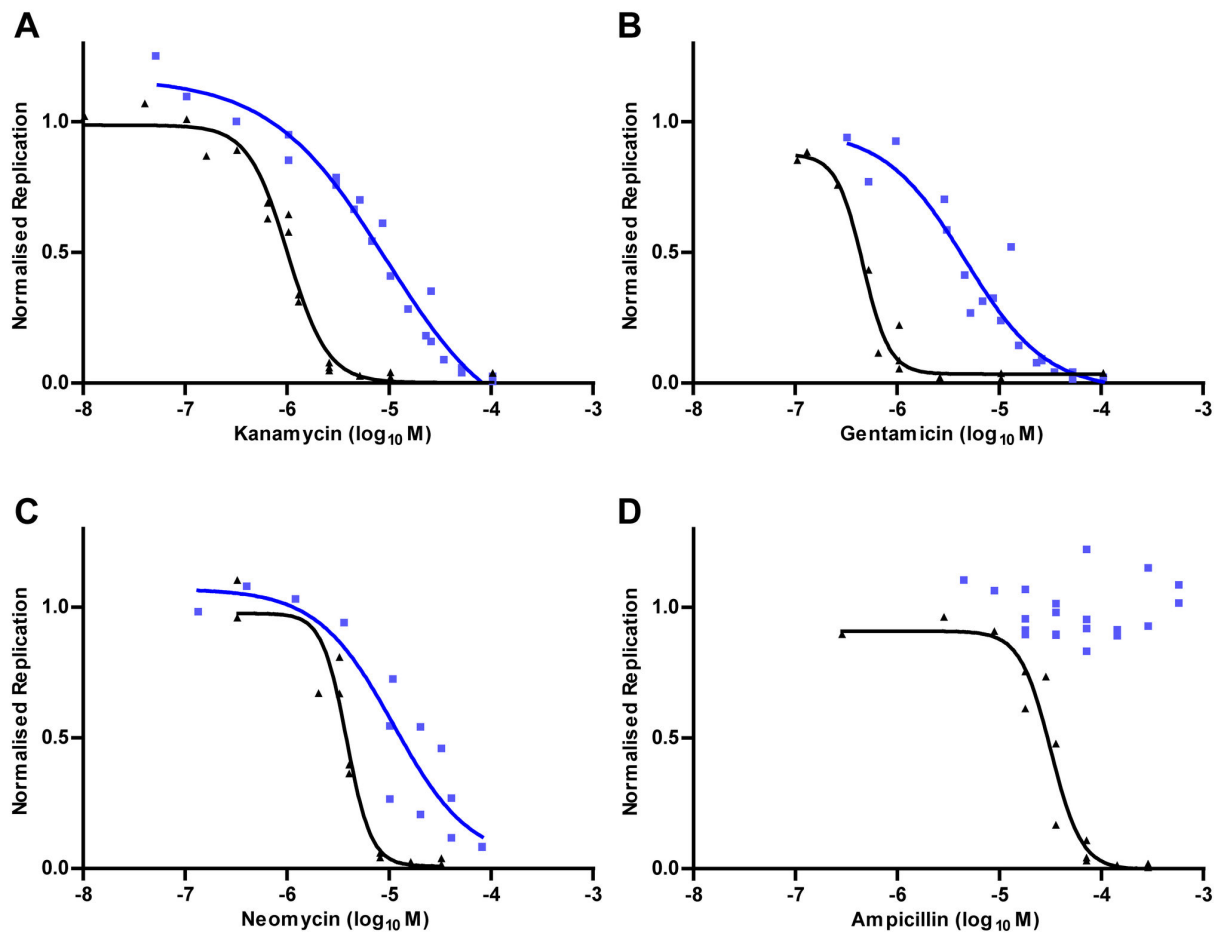
Sensitivity of intra- or extracellular *L. pneumophila* to antibiotics. **A**-**D**. *L. pneumophila* infecting *A* *. castellanii* (blue squares) or growing in AYE broth (black triangles) was treated with the antibiotics (**A**) kanamycin, (**B**) gentamicin, (**C**) neomycin or (**D**) ampicillin. Replication values were normalised between 0 (background / no bacteria) and 1 (replication of vehicle control), and dose-response curves were constructed from at least three independent experiments, with calculated lines of best fit.

The suitability of the *L. pneumophila /* *A* *. castellanii* assay for drug screening was further assessed by examining the effects of antibiotics. Infected cells were treated with gentamicin, ampicillin, kanamycin or neomycin at a range of concentrations, and replication was allowed to proceed until the vehicle control had entered stationary growth phase. Fluorescence data were normalized between 0 (background / no bacteria) and 1 (vehicle control), and dose-response curves were plotted (Figure 2). For comparison the dose-response inhibition for extracellular growth (i.e. in broth) for the same antibiotics was determined and used to calculate the Cell Index, a measure of the degree of protection afforded to intracellular bacteria (Table 1). Intracellular bacteria were more resistant than extracellular to all antibiotics tested, an effect which was most apparent with ampicillin.

**Table 1:** IC_50_ values of antibiotics for intra- and extracellular *L. pneumophila*.

**Antibiotic**	**Intracellular**		**Extracellular**		**Cell index** ^1^
	**IC_50_** (µM)	**95% CI** (µM)	**IC_50_** (µM)	**95% CI** (µM)	
**kanamycin**	9.3	5-17	1	0.9-1.2	9.3
**gentamicin**	4.6	2.4-8.8	0.46	0.4-0.6	10
**neomycin**	11	3.5-35	3.8	3.1-4.7	2.9
**ampicillin**	N/C ^2^	N/C	31	24-41	N/C

^1^ Intracellular *versus* extracellular

^2^ N/C, not calculable

**Figure 3:**
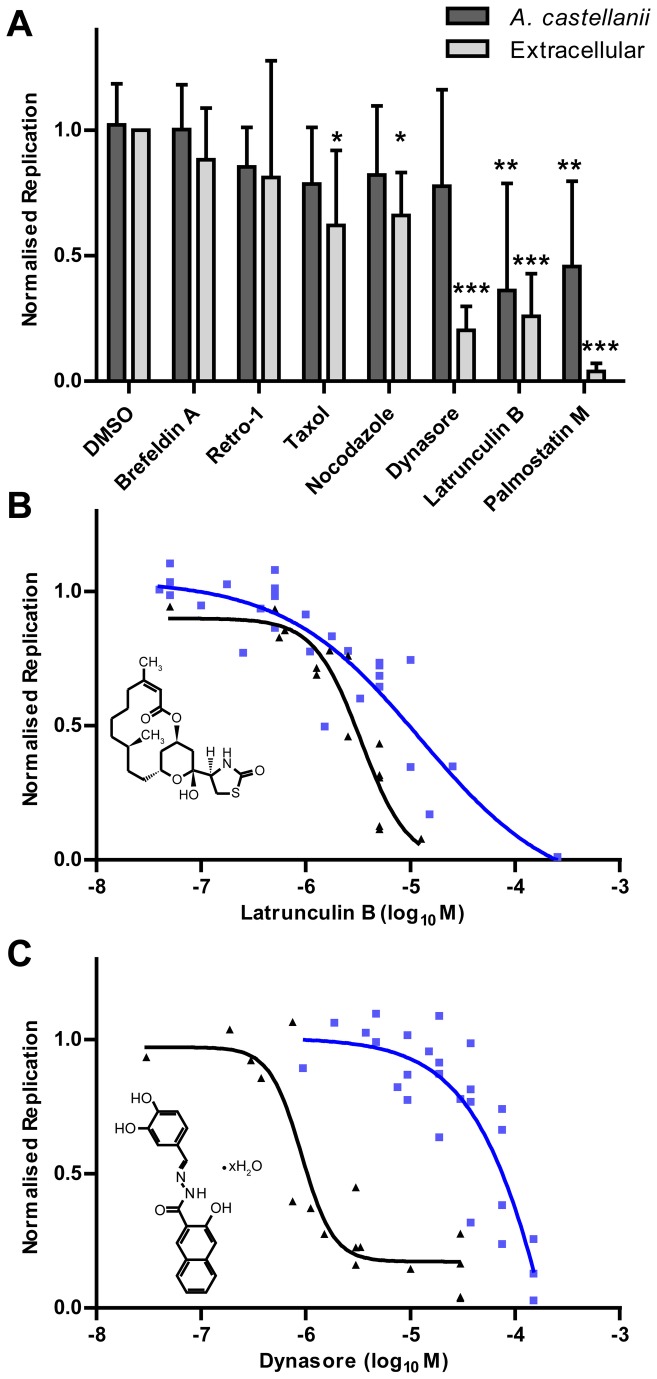
Sensitivity of intra- or extracellular *L. pneumophila* to host-targeting compounds. **A**. The growth of *L. pneumophila* in the presence of various compounds (10 µM) was determined for both intracellular ( *A* *. castellanii* ) and extracellular replication. The graphs indicate the mean and 95% confidence intervals of OD_600_ or fluorescence measurements normalised between 0 (background / no bacteria) and 1 (vehicle control) (* *p* < 0.05, ** *p* < 0.01, *** *p* < 0.001 (*t*-test) compared to DMSO control). B-C. Dose-response curves showing inhibition of intracellular (blue) and extracellular (black) replication by (**B**) latrunculin B, an inhibitor of actin polymerisation, or (C) dynasore, an inhibitor of dynamin-mediated vesicle scission. All graphs indicate the combined averages of at least 3 independent experiments.

Next, the effects of compounds predicted to affect the host cell rather than the bacteria were examined. These compounds included taxol and nocodazole (targeting microtubules), latrunculin B (an actin polymerisation inhibitor), brefeldin A, dynasore and retro-1 (interfering with vesicle transport), as well as palmostatin M and B (blocking Ras signalling) (Figure 3). Of these, only latrunculin B, dynasore and palmostatin M caused significant reduction in either intra- or extra-cellular growth of *L. pneumophila* at 10 µM (Figure 3A). Taxol, nocodazole and brefeldin A inhibited intra- as well as extracellular replication of *L. pneumophila*, but only at high concentrations (Figure S2). The dose-response curves of latrunculin B and dynasore were examined in more detail to identify IC_50_ values (Figure 3B, C). Latrunculin B showed a low IC_50_ (12 µM) for intracellular growth compared to dynasore (IC_50_: 420 µM) (Table 2). Surprisingly, however, latrunculin B and in particular dynasore exhibited IC_50_ values in the low micromolar range for extracellular growth and thus appeared to be effective antibiotics, rather than anti-virulence compounds. As observed with the classical antibiotics, intracellular bacteria were significantly less sensitive (> 3-fold) to these growth-inhibiting compounds.

**Table 2:** IC_50_ values of host-targeting compounds for intra- and extracellular *L. pneumophila*.

**Compound**	**Intracellular**		**Extracellular**		**Cell index** ^1^
	**IC_50_** (µM)	**95% CI** (µM)	**IC_50_** (µM)	**95% CI** (µM)	
**latrunculin B**	12	2.4-62	3.4	1.8-6.4	3.6
**dynasore**	420	V. wide	0.9	0.6-1.5	470

^1^ Intracellular *versus* extracellular

### Palmostatin M selectively inhibits growth of *Legionella* and *Mycobacterium* species

**Figure 4:**
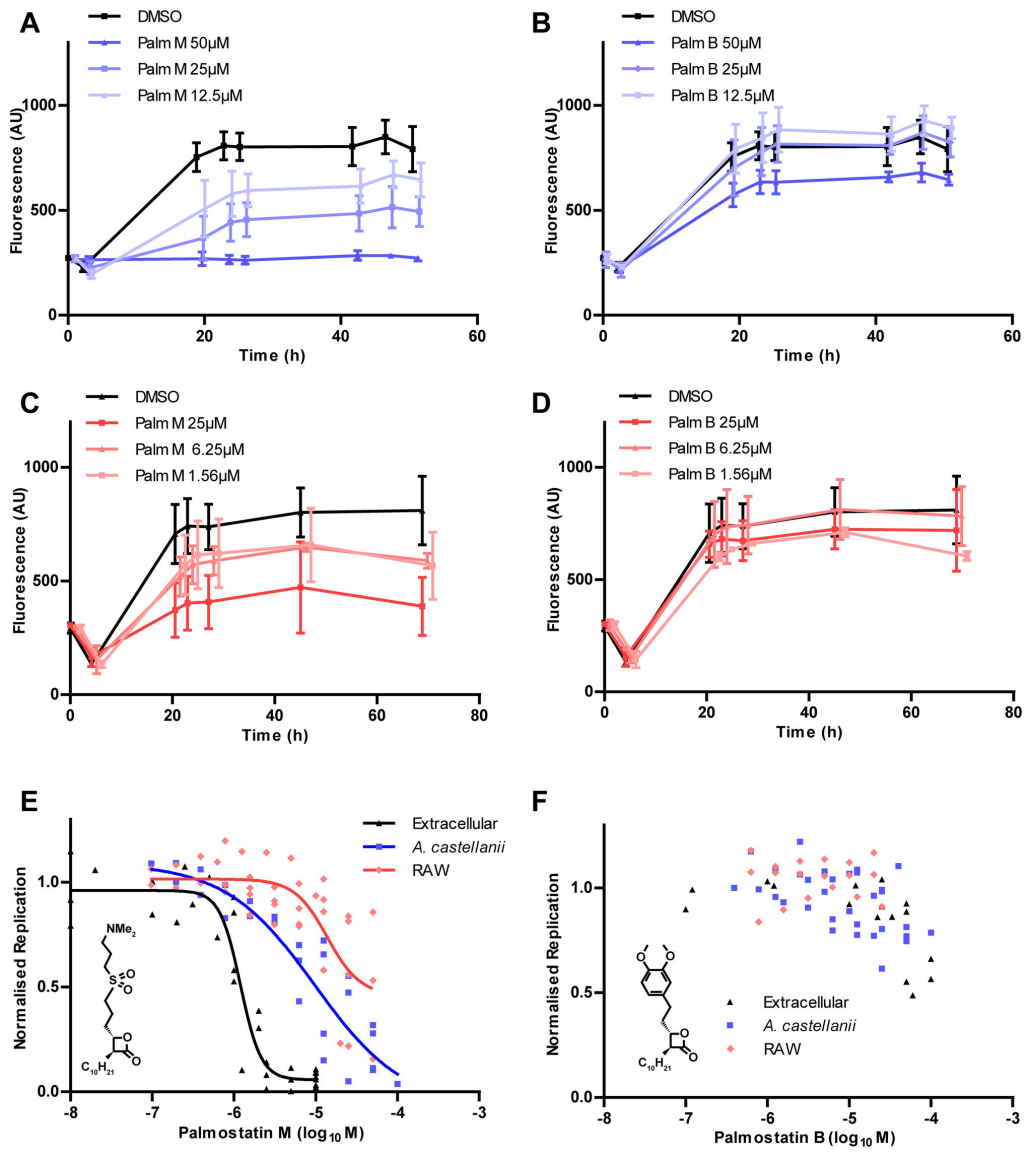
Sensitivity of intra- or extracellular *L. pneumophila* to palmostatin M and B. **A**-**F**. Time course of *L. pneumophila* infecting *A* *. castellanii* (**A**, **B**) or RAW 264.7 macrophages (**C**, **D**) in the presence of palmostatin M (**A**, **C**) or palmostatin B (**B**, **D**). Time courses are representative experiments showing mean and 95% confidence intervals of three wells. Dose-response curves of palmostatin M (**E**) or palmostatin B (**F**) indicate that palmostatin M inhibits replication of *L. pneumophila* in AYE (black triangles), *A* *. castellanii* (blue squares) and RAW 264.7 macrophages (red diamonds). Curves indicate averages and calculated curves for data from at least 3 independent experiments, normalised between 0 (no replication) and 1 (vehicle).

To characterize the apparent antibacterial activity of presumably host cell-active compounds, we focused on palmostatin M for further analysis. Palmostatin M inhibits the eukaryotic hydrolase enzymes APT1 and APT2 by blocking their Ras GTPase depalmitoylation activity and in turn altering Ras GTPase localization and activity [26,27]. The β-lactone Palmostatin M may also have off-target effects on bacterial serine hydrolases with a similar active site. Palmostatin M inhibited the intracellular growth of *L. pneumophila* in a dose-dependent manner in *A* *. castellanii* (Figure 4A) as well as in RAW 264.7 macrophages (Figure 4C). In contrast, the related β-lactone-based APT inhibitor palmostatin B did not inhibit intracellular replication in *A* *. castellanii* (Figure 4B) or in macrophages (Figure 4D). A secondary analysis for toxicity indicated that the palmostatin compounds did not affect the growth or viability of *A* *. castellanii* (Figure S2). Direct comparison revealed that extracellular *L. pneumophila* was much more sensitive to palmostatin M than intracellular bacteria (Figure 4E; Table 3). The extracellular replication of *L. pneumophila* was slightly inhibited by very high concentrations of palmostatin B (Figure 4F). These experiments demonstrate that palmostatin M inhibits the growth of *L. pneumophila* in host cells as well as in broth, indicating an as-yet unrecognized potential as an antibiotic for this compound.

**Table 3:** IC_50_ values of palmostatins for intra- and extracellular *L. pneumophila*.

	**palmostatin M**			**palmostatin B**			**Cell index** ^1^
	**IC_50_** (µM)	**95% CI** (µM)		**IC_50_** (µM)	**95% CI** (µM)		
** *A* *. castellanii* **	9.8	1.9-50		18	0.3-240		8.2 (palm M)
**RAW 264.7**	14	5.9-32		N/C ^2^	N/C		11.6 (palm M)
**Extracellular**	1.2	1-1.5		72	N/C		N/C

^1^ Intracellular *versus* extracellular

^2^ N/C: not calculable

**Figure 5:**
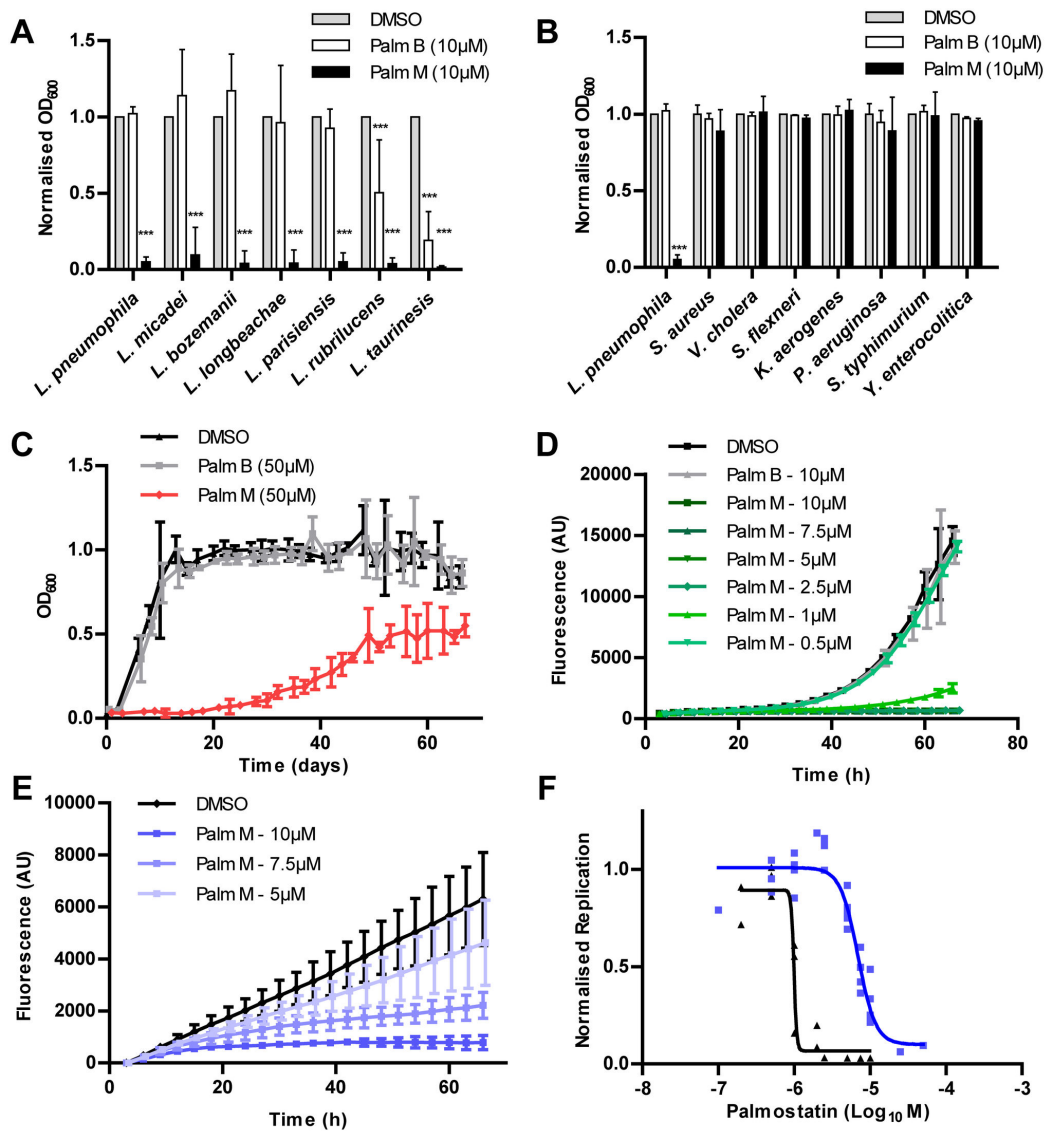
Species specificity of bacterial growth inhibition by palmostatin M. **A**-**E**. Extracellular growth of (**A**) *Legionella* species (in AYE), (**B**) the bacterial species indicated (in LB), (**C**) *Mycobacterium tuberculosis* (in 7H9), and (**D**) *Mycobacterium* *marinum* (in 7H9) or (**E**) *M. marinum* infecting *A* *. castellanii* (MOI 10) was determined in the presence of 10 µM palmostatin M or palmostatin B. DMSO was used as a vehicle control. Palmostatin M specifically inhibited extracellular growth of the *Legionella* and *Mycobacterium* species tested. **F**. Dose response curve showing inhibition of intracellular ( *A* *. castellanii* ) (blue) and extracellular (black) replication of *M. marinum* by palmostatin M. Graphs indicates mean, error bars show 95% confidence intervals of at least 3 or 2 (*M*. *tuberculosis*) independent experiments (*** *p* < 0.001 (*t*-test) compared to DMSO control).

Next, species specificity was examined by determining the effect of 10 µM palmostatin M or palmostatin B on the extracellular growth of various *Legionella* species (Figure 5A). Palmostatin M prevented the growth of all *Legionella* species tested, whereas palmostatin B was ineffective against all species except *L* *. taurensis* . Further testing of palmostatin M or palmostatin B against a range of bacterial pathogens showed that their growth was unaffected by palmostatin M or palmostatin B (Figure 5B). Thus, palmostatin M selectively inhibits the growth of *Legionella* spp. in a group of various Gram-positive or Gram-negative, extra- or intracellular, vacuolar or cytoplasmic pathogens.

Although palmostatin M appeared specific to *Legionella* , another vacuolar pathogen is *Mycobacterium tuberculosis*, which - due to its biosafety level 3 requirements - was not originally tested. Interestingly, similar to *Legionella* spp. *Mycobacterium tuberculosis* grown in broth was efficiently inhibited by palmostatin M but not palmostatin B (Figure 5C). Since *M. tuberculosis* and *L. pneumophila* are both intracellular vacuolar pathogens, we determined whether palmostatin M could inhibit intracellular replication of a *Mycobacterium* spp. Due to a lack of suitable hosts for *M. tuberculosis* the closely related fish and opportunistic human pathogen *Mycobacterium* *marinum* was used, which replicates in *D. discoideum* [28,29] and *A* *. castellanii* amoebae (Kicka S. et al., manuscript in preparation). As with *M. tuberculosis*, palmostatin M but not palmostatin B efficiently inhibited the extracellular growth of *M. marinum* (Figure 5D), as well as the intracellular growth of *M. marinum* within *A* *. castellanii* (Figure 5E). Finally, dose-response curves indicated that, as with *L. pneumophila*, intracellular residence protected *M. marinum* against palmostatin M (Figure 5F). Thus, the β-lactone compound palmostatin M, but not the related compound palmostatin B, prevented both intra- and extracellular replication of *Legionella* and *Mycobacterium* species.

**Figure 6:**
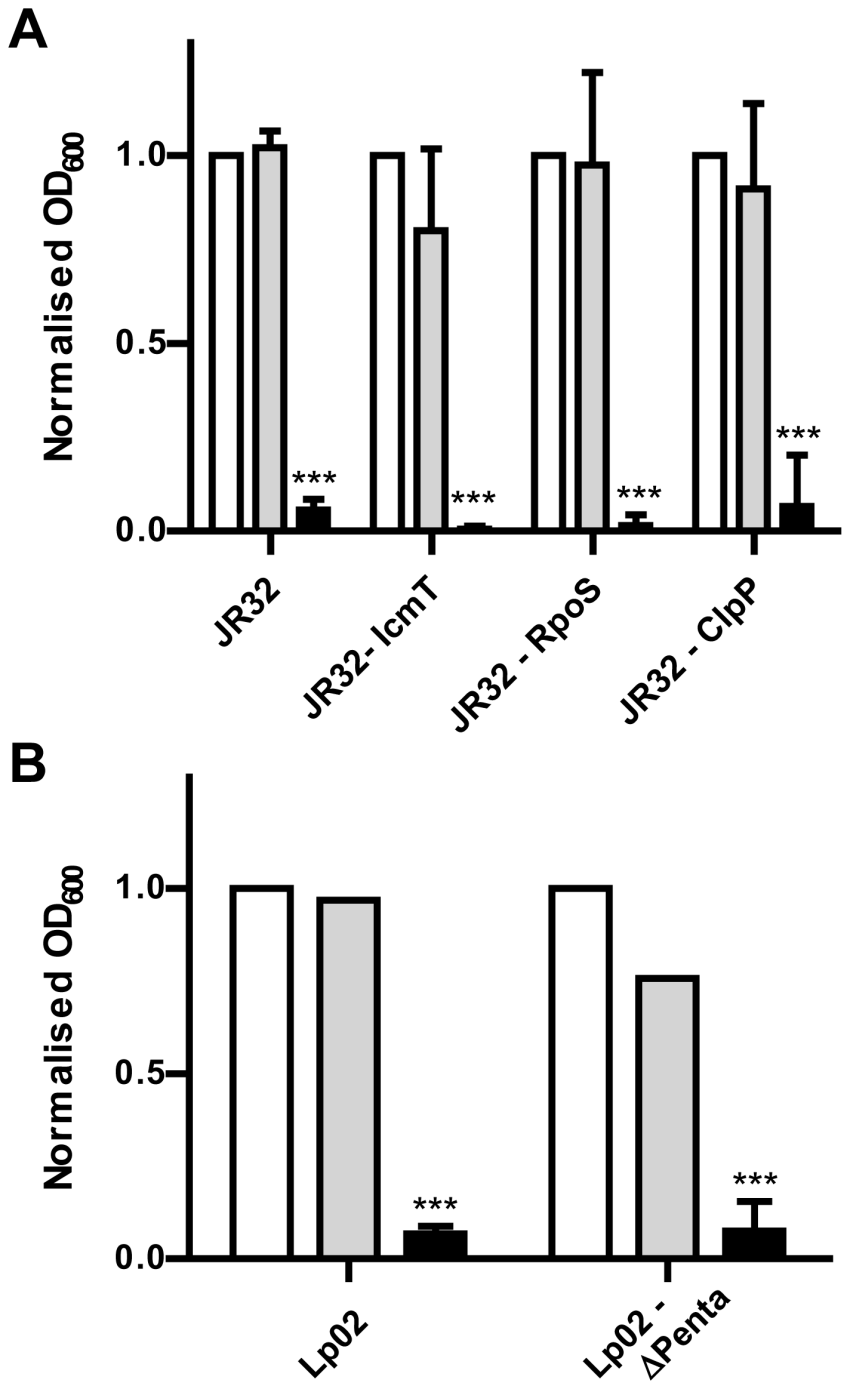
Growth inhibition of *L. pneumophila* mutant strains by palmostatin M. The extracellular growth of *L. pneumophila* deletion mutants was determined in AYE medium in the presence of 10 µM palmostatin M (black bars), palmostatin B (grey), or DMSO (white). Palmostatin M equally affected *L. pneumophila* lacking (**A**) IcmT (component of Icm/Dot T4SS), RpoS (alternative sigma factor), or ClpP (catalytic subunit of ClpAP protease); as well as (**B**) 18% of the genome (ΔPenta). The OD_600_ was measured after 24 h and normalized to the DMSO control. Graph indicates the mean and 95% confidence intervals of at least 3 experiments (*** *p* < 0.001 (*t*-test) compared to DMSO control).

In attempts to identify the target of palmostatin M, the effect of palmostatin M and palmostatin B on the growth of a several defined deletion mutants of *L. pneumophila* was tested (Figure 6A, B). Similar to wild type *L. pneumophila*, palmostatin M but not palmostatin B inhibited growth of mutant strains lacking the T4SS subunit IcmT [23], the alternative sigma factor RpoS [24], the ClpP subunit of the ClpAP self-compartmentalizing protease [30], or approximately 18.5% of the genome [31]. The growth of these mutants was inhibited to a similar extent as wild-type *L. pneumophila*, indicating that the missing bacterial factors likely do not play a role regarding the mode of action of palmostatin M.

## Discussion

This study details the development of an assay to continuously monitor by fluorescence the intracellular replication of *L. pneumophila* within three phagocytic model host organisms, and shows how chemical inhibitors of growth can be characterised by this system. Previous attempts to develop new antibiotics via HTS have not had significant success [14], partly due to the difficulty of converting compounds which affect isolated bacterial factors into those with the same effect in live bacteria. The advantage of assaying compounds in a *L. pneumophila /* *A* *. castellanii* system is the ability to detect compounds which target the infection and intracellular replication process. However, this approach requires a robust secondary screen for compounds that simply kill the host cell. In this study the Alamar Blue viability assay was used to determine the cytotoxicity of compounds against *A* *. castellanii* host cells.

The complexity introduced by screening small molecule libraries against two interacting organisms raises the challenge to obtain reliable and reproducible results. Experimental values were used to calculate the Z-factor, a measure of the sensitivity and reliability of an assay [32]. The final calculated value of 0.61 indicates a satisfactorily robust assay, and is comparable to other screens that have been performed on intracellular pathogen replication [33]. In our assay, a higher variation between replicates was generally observed when compared to extracellular growth (e.g. Figure 3A), and thus it was necessary that all intracellular replication assays were performed in at least triplicate independent experiments. Thus the *L. pneumophila /* *A* *. castellanii* assay appears suitable for screening compound libraries at a medium-throughput scale (in the range of 10,000 compounds), however the greater variability and the challenges involved in scaling beyond 96-well plates are likely to prevent screening of larger libraries.

The process of intracellular replication was successfully quantitated in three host cells, *A* *. castellanii* , *D. discoideum* and RAW 264.7 macrophages. Whereas *A* *. castellanii* and *D. discoideum* behaved similarly, the macrophages showed higher final replication levels when infected at low initial MOIs. This might be due to an increased cytotoxicity of *L. pneumophila* against macrophages at higher MOIs, or due to a lower uptake efficiency allowing further replication of uninfected macrophages during the assay; in effect working as a multiple rather than single-round assay. The requirement to incubate *D. discoideum* at the lower temperature of 25°C led to a significantly reduced rate of *L. pneumophila* replication, and thus, this amoeba was less suitable for screening than the more thermo-tolerant *A* *. castellanii* . *D. discoideum* is a commonly used model system for *L. pneumophila* infection, in large part due to the ease of genetic manipulation [34,35,36]. Therefore, while unsuitable for primary screening, *D. discoideum* allows later confirmation of suspected target proteins, for example those implicated in uptake, vesicle trafficking and replication of *L. pneumophila*, such as RtoA, PI3Ks, or Dd5P4 [36,37,38].

Initial validation using antibiotics showed that, as expected, the compounds prevented the extracellular growth of *L. pneumophila*. The dose-response curve was steeper for extracellular bacteria when compared to intracellular, whereas intracellular bacteria had a greater than 3-fold higher resistance against antibiotics. This suggests a greater overall efficacy of the antibiotics and/or a more uniformly sensitive population of extracellular bacteria. The findings are also in agreement with previous work, which has shown increased antibiotic resistance for intracellular bacteria [39] and for bacteria emerging from host cells [11].

Ampicillin did not affect intracellular replication, possibly due to expression of the *Legionella* β-lactamase gene, *loxA* [40], in combination with other genes differentially expressed in intracellular bacteria. Alternatively or additionally, access of the antibiotic to the phagocytic vacuole might be severely impaired, a mechanism that seems to apply to the human monocytic THP1 cell line [41]. In contrast, intracellular growth of *L. pneumophila* was inhibited by gentamicin, a nominally cell-membrane-impermeable antibiotic. This suggests that the interaction between the LCV and the outer milieu is more permissive than expected. The finding correlates with previous work, demonstrating that *Legionella* -infected human lung fibroblasts can be cured with gentamicin, thus indicating the accessibility of the pathogen vacuole to this antibiotic [42].

We also tested small molecules expected to affect bacteria-host cell interactions but not *L. pneumophila* directly, including compounds that target microtubules (taxol, nocodazole), actin polymerisation (latrunculin B), vesicle transport (brefeldin A [43], dynasore [44], retro-1 [45]) or Ras GTPase localisation (palmostatin M, an inhibitor of Ras depalmitoylation [26]). These compounds have been developed for mammalian cells, which may explain their moderate effect on replication within *A* *. castellanii* . Surprisingly, all molecules with an effect on *L. pneumophila* replication within amoebae were also able to prevent bacterial growth in broth, the most notable examples being dynasore and palmostatin M. Previous work has shown an antibacterial activity for minor structural variants of latrunculin B [46]; however the potent antibiotic activity of dynasore and palmostatin M has not been reported before. The antibiotic effects were also significantly more apparent in broth-grown *L. pneumophila* than intracellular bacteria, in line with previous observations that intracellular localization protects bacteria from otherwise toxic compounds [8,41].

Interestingly, our results indicate a specific antibacterial activity of palmostatin M, which inhibited the growth of all tested *Legionella* and *Mycobacterium* species, while having no effect on the growth rates of a wide range of other bacterial genera. A potential target protein of palmostatin M is the “self-compartmentalizing” bacterial protease ClpP, which has been shown to bind compounds with β-lactone rings such as palmostatin M in *Staphylococcus aureus* [47,48]. ClpP is not essential for *L. pneumophila*, but a deletion mutant strain exhibits slower growth rates and is unable to replicate in *A* *. castellanii* [30]. By contrast, the two homologues of ClpP in *M. tuberculosis* are essential for the survival of the bacteria, and have been validated as a target for acyldepsipeptides [49]. While *L. pneumophila* ClpP knockouts were equally affected by palmostatin M, it is possible that a homologous protein is the actual target. BLAST searches of the *L. pneumophila* genome revealed the existence of a homologue of human acyl-protein thioesterase 1, the original target of palmostatin M and B. This gene, *lpg0369*, is annotated as a hypothetical carboxylesterase/phospholipase (37% identity, 57% similarity), but as no analogue appears to exist in mycobacteria, it is unlikely to represent a common target in both *Legionella* and *Mycobacterium* spp. Further pull-down attempts with structural homologues of palmostatin M may provide more information.

Despite the current lack of knowledge regarding the palmostatin M target, this molecule represents a starting point for the development of potentially novel antibiotics. Inhibition of intracellular growth is a vital property for any compound developed against *Legionella* and *Mycobacterium* spp., both of which spend a significant and important part of their life cycle within eukaryotic cells [50,51]. The lack of broad spectrum antibiotic activity of palmostatin M does not preclude its development as a targeted antibiotic; indeed this may be preferable when coupled with improved diagnostic methods. However, one major pitfall of palmostatin M as an antibiotic is its previously established effect on APT1 (IC_50_ < 5 nM in biochemical assays [52]), and thus on Ras localisation and signalling. Ras GTPase is involved in a wide range of cellular processes, including cell proliferation [53]. An IC_50_ < 5 nM is about 1,000 × (*L. pneumophila*) or 100 × (*M. marinum*) lower than that observed to inhibit the replication of extracellular bacteria. Thus, careful screening of structural homologues is required to find compounds with specificity towards the bacterial targets rather than human acyl-protein thioesterases.

In summary, this study presents the development of a sensitive, discriminatory and continuous assay for the intracellular replication of *L. pneumophila* within the model host amoeba, *A* *. castellanii* . The assay was validated by determining the inhibitory concentrations required for antibiotics and a range of host-cell acting compounds. In this process an unexpected antibacterial property of the APT1 inhibitor palmostatin M was discovered. The antibacterial activity of palmostatin M appears to be specific to members of the genera *Legionella* and *Mycobacterium* . The assay thus shows promise for medium-throughput screening of libraries of small molecule compounds which affect bacterial intracellular replication.

## Materials and Methods

### Bacterial strains, growth condition and reagents

The bacterial strains used in this study are listed in Table S1. *L. pneumophila* JR32 Δ*clpP* was a kind gift of the Dr. Yong-jun Lu, Sun Yat Sen University, China [30]. The *L. pneumophila* pentuple deletion mutant was a kind gift of Dr. Ralph Isberg, Tufts University, USA [31]. The bacteria (except *M. tuberculosis*) were resuspended from plates in appropriate growth medium (ACES Yeast Extract (AYE [54]) or Luria Broth (LB)) and diluted to a starting OD_600_ of 0.01. Compounds were added to these cultures such that the maximal DMSO concentration was 0.1%. Cultures were grown overnight (or over several weeks in the case of *M. tuberculosis*) and the OD_600_ measured. Data were normalized to DMSO controls and graphs compiled from the average value of at least 3 separate experiments.

Growth of *M. marinum* was assayed in BBL, Middlebrook 7H9 broth by seeding 1 × 10^5^ bacteria into a 96-well plate with or without compounds, and replication was followed for up to 48 h by monitoring the OD_600_. *M. tuberculosis* suspensions were prepared at an OD_600_ of 0.3 in BBL, Middlebrook 7H9 broth with glycerol (BD Bioscience). Triplicate cultures were prepared as a 500 µl suspension in 5 ml 7H9 broth in the presence of DMSO or 50 µM palmostatin M. The cultures were incubated in tubes at 37°C and the OD_600_ was measured every 2-3 days.

Reagents and compounds were from Sigma-Aldrich, if not indicated otherwise, whereas palmostatin B and M were provided by Dr. Christian Hedberg, Max Planck Institute of Molecular Physiology, Dortmund.

### Intracellular replication of *L. pneumophila*

*A* *. castellanii* were cultured in PYG medium [21] and split the day prior to infection such that 2 × 10^4^ cells were present in each well of a 96-well plate (Cell Carrier, black, transparent bottom from PerkinElmer). Cultures of *L. pneumophila* harbouring the GFP-producing plasmid pNT-28 [19] were resuspended from plate to a starting OD_600_ of 0.1 in AYE medium, and grown overnight on a rotating wheel at 37°C to an OD_600_ of 3. Bacteria were diluted in LoFlo medium (ForMedium) such that each well contained 8 × 10^5^ bacteria (MOI 20). Infections were synchronised by centrifugation at 1500 rpm for 10 min. Compounds were added to at least triplicate wells during or after infection depending on the susceptibility time frame being assessed (see Data Analysis for more details). Infected cultures were incubated in a 30°C incubator, and the GFP fluorescence was measured by a plate spectrophotometer at appropriate intervals (Optima FluoStar, BMG Labtech). Time courses were constructed and data was used to determine the effect of compounds versus vehicle control.

Comparison of *L. pneumophila* deletion mutant strains was performed in a similar manner. To control for any changes in GFP fluorescence between strains the fluorescence/OD_600_ was determined for each overnight culture and used to normalise later measurements. *D. discoideum* was cultivated in HL-5 medium, and infections were performed identically as *A* *. castellanii* with the sole change that infected amoebae were incubated at 25°C due to their thermosensitivity.

RAW 264.7 macrophages (ATCC: Tib-71, lab collection) were cultured in RPMI medium containing 5% FCS (Gibco). Cells were seeded at 8 × 10^4^ cells/well in a 96-well plate, to allow for the apparently lower infection efficiency. Infection was performed as with *A* *. castellanii* ; however, bacteria and compounds were diluted in RPMI. The medium was changed 2 h post infection to ensure removal of non-phagocytosed bacteria.

### Intracellular replication of *M. marinum*

*A* *. castellanii* cells in PYG were allowed to settle on 10 cm dishes at 80% confluence. *M. marinum* (msp12::GFP, a kind gift from L. Ramakrishnan) were grown to a final OD_600_ of 1 (5 × 10^8^ bacteria/ml) in flasks containing 7H9 medium supplemented with OADC (Oleic Acid Albumin Dextrose Complex), Tween 80, glycerol and kanamycin, as well as beads to assist growth. *M. marinum* were washed twice with PYG medium, and residual clumps were eliminated by passage through a 3 µm filter.

For the infection, the bacteria were homogeneously added onto 10 cm dishes containing approximately 2 × 10^7^ adherent *A* *. castellanii* (MOI 10). The dishes were centrifuged twice at 500 × *g* for 10 min and turned 180 degrees in-between the two centrifugation steps to avoid accumulation of cells and bacteria on one side of the dish. The cells were incubated at 25°C for 10-20 min, and excess extracellular bacteria were carefully removed by washing with fresh PYG. Amikacin (10 µM) was added at a concentration that completely inhibited extracellular bacterial growth without affecting intracellular growth. A 96-well plate suitable for fluorescence plate reading was filled with 100-200 µl of a suspension of infected cells; each well containing 2.5 × 10^4^ *A* *. castellanii* cells and incubated at 25°C. Fluorescence was recorded in a plate reader.

### Cytotoxicity Assay

Cytotoxicity of compounds against *A* *. castellanii* was determined using Alamar Blue reagent (Life Technologies). To mimic the conditions found in the intracellular replication assay, 96-well plates were set up as previously described and uninfected triplicate wells were treated with compounds in 100 µl LoFlo media. Plates were incubated at 30°C for 24 h, after which 10 µl Alamar Blue reagent was added, and plates incubated for a further 3 h. The fluorescence at 595 nm was measured, and data normalized between 1 (treatment with LoFlo alone) and 0 (SDS, total lysis of the cells). Means from each individual experiment were then combined for analysis.

### Data analysis

Data analysis was performed in Microsoft Excel and Graph-pad Prism 5. To compare the effect of compound treatment on intracellular replication, fluorescence values were taken from the first time point following entry to stationary phase. The results were then normalised such that media-only wells (no bacteria) were 0, whereas vehicle-treated wells were 1 (normal replication). The average of the replicate wells (minimum 3 per plate) was then plotted as dose-response curves, such that each individual point represented the average of a single experiment. Compound treatments were repeated a minimum of 3 times to control for the increased variability of bacteria-host cell interactions. Lines of best fit and associated IC_50_ values were calculated for each dose-response curve using the non-linear fit function of Prism 5 (log inhibitor versus response, variable slope). The Cell Index was calculated as the fold-increase in intracellular versus extracellular IC_50_.

## Supporting Information

Figure S1 **Replication of *L. pneumophila* within *Dictyostelium discoideum*.** **A**-**B**. *D. discoideum* amoeba were infected in 96-well plates with GFP-producing (**A**) wild-type *L. pneumophila* at the MOIs indicated, or (**B**) wild-type *L. pneumophila* or mutants (MOI 10), and the progress of intracellular growth was followed by fluorescence measurement over 7 days using a microtiter plate reader. Intracellular replication of *L. pneumophila* in *D. discoideum* occurs over a longer time scale than that observed in *A* *. castellanii* , and the deletion mutants Δ*icmT*, Δ*flaA* or Δ*rpoS* also show replication defects in *D. discoideum*. **C**. Replication of *L. pneumophila* deletion mutants in *A* *. castellanii* was followed over the course of two days by CFU assay. Time course shows mean and standard deviation of the same representative experiment shown in Figure 1C and 1D.
(TIF)

Figure S2 **Dose-response curves of host-targeting compounds.** Dose-response curves of (**A**) taxol, (**B**) nocodazole and (**C**) brefeldin A, showing the effect on intracellular (* A. castellanii *) (blue) and extracellular replication (black). Graphs indicate combined results from at least 3 independent experiments. **D**. Cytotoxicity assay following 24 h treatment of *A* *. castellanii* with 10 µM palmostatin A or palmostatin B, as assayed by an Alamar Blue viability assay. No difference in replication of the amoebae was observed. Graph indicates mean and 95% confidence interval of 5 independent experiments.
(TIF)

Table S1 **Bacterial strains used in this study.**
(DOCX)
