# Supplementary figures and images for: Exploring Anti-Bacterial Compounds against Intracellular Legionella
Source: PLoS One. 2013 Sep 13;8(9):e74813. doi: 10.1371/journal.pone.0074813 (PMC3772892; doi:10.1371/journal.pone.0074813)

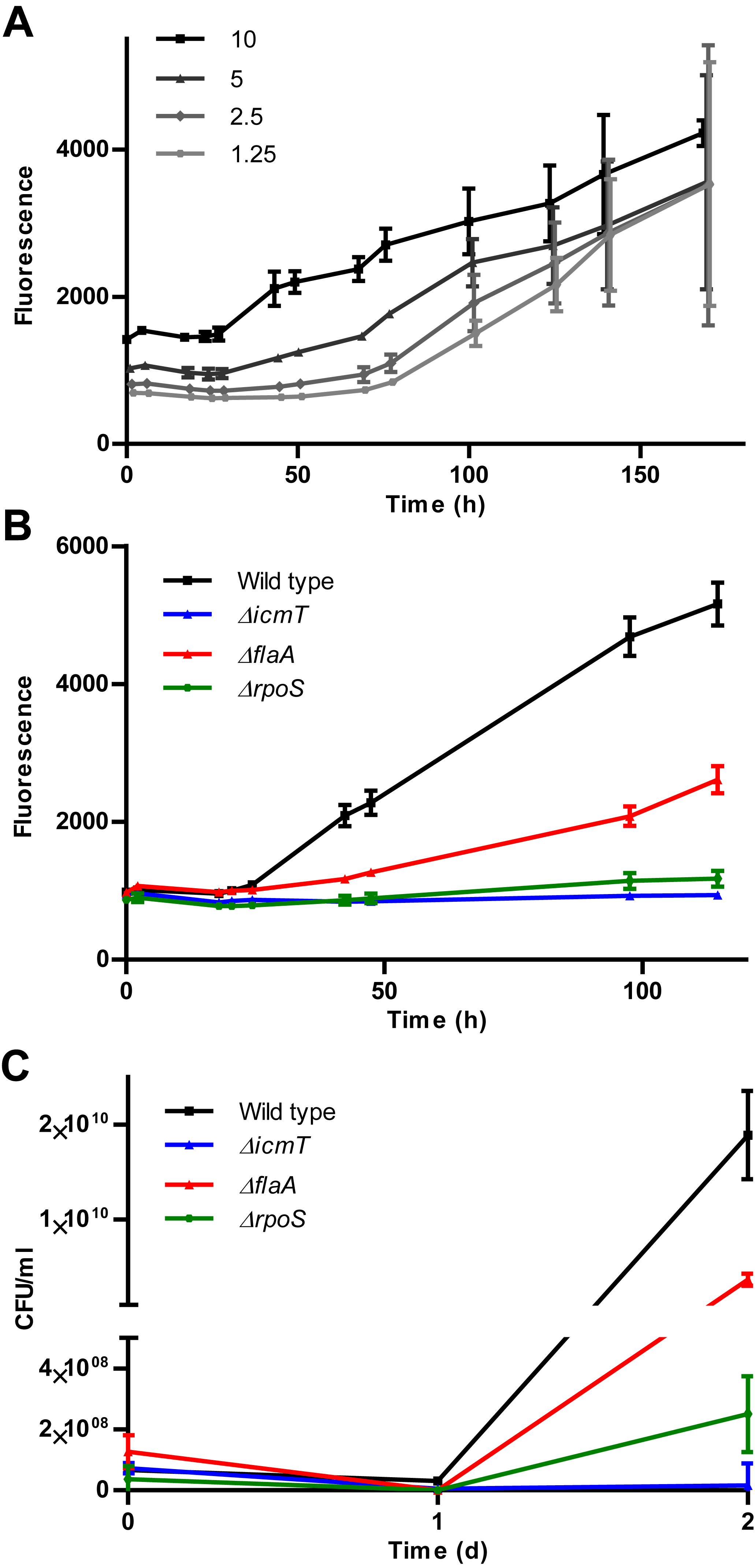

Supplement: Figure S1 — Replication of L. pneumophila within Dictyostelium discoideum. A-B. D. discoideum amoeba were infected in 96-well plates with GFP-producing (A) wild-type L. pneumophila at the MOIs indicated, or (B) wild-type L. pneumophila or mutants (MOI 10), and the progress of intracellular growth was followed by fluorescence measurement over 7 days using a microtiter plate reader. Intracellular replication of L. pneumophila in D. discoideum occurs over a longer time scale than that observed in A . castellanii , and the deletion mutants ΔicmT, ΔflaA or ΔrpoS also show replication defects in D. discoideum. C. Replication of L. pneumophila deletion mutants in A . castellanii was followed over the course of two days by CFU assay. Time course shows mean and standard deviation of the same representative experiment shown in Figure 1C and 1D. (TIF) [file pone.0074813.s001.tif]

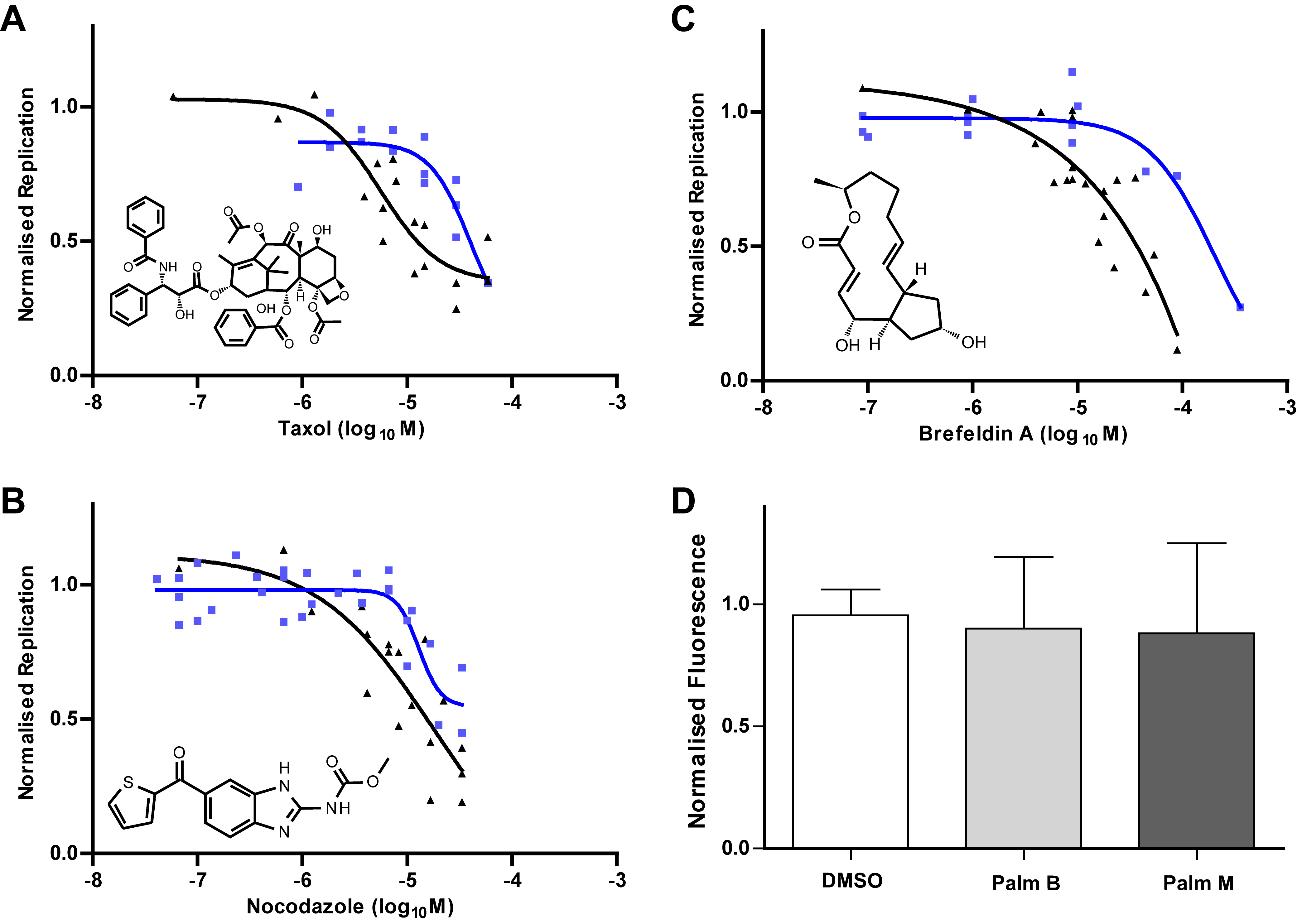

Supplement: Figure S2 — Dose-response curves of host-targeting compounds. Dose-response curves of (A) taxol, (B) nocodazole and (C) brefeldin A, showing the effect on intracellular ( A. castellanii ) (blue) and extracellular replication (black). Graphs indicate combined results from at least 3 independent experiments. D. Cytotoxicity assay following 24 h treatment of A . castellanii with 10 µM palmostatin A or palmostatin B, as assayed by an Alamar Blue viability assay. No difference in replication of the amoebae was observed. Graph indicates mean and 95% confidence interval of 5 independent experiments. (TIF) [file pone.0074813.s002.tif]
