# Supplementary material for: Exploring Anti-Bacterial Compounds against Intracellular Legionella
Source: PLoS One. 2013 Sep 13;8(9):e74813. doi: 10.1371/journal.pone.0074813 (PMC3772892; doi:10.1371/journal.pone.0074813)
Supplement: Table S1 — Bacterial strains used in this study. (DOCX) [file pone.0074813.s003.docx]

**Table S1: Bacterial strains used in this study.**

| **Bacterial strain** | **Relevant properties ^a^** | **Reference** |
| --- | --- | --- |
| *L. pneumophila* |  |  |
| JR32 | *L. pneumophila* serogroup 1, strain Philadelphia-1, salt-sensitive isolate of AM511 | [[1](#_ENREF_1)] |
| Δ*icmT* (GS3011) | JR32 *icmT3011*::Km | [[2](#_ENREF_2)] |
| Δ*clpP* | JR32 *clpP*::Km | [[3](#_ENREF_3)] |
| Δ*flaA* | *L. pneumophila* serogroup 1, strain Corby *flaA*::Km | [[4](#_ENREF_4)] |
| Δ*rpoS* (LM1376) | JR32 *rpoS4*::Tn*903*dIIGm | [[5](#_ENREF_5)] |
| Lp02 | *L. pneumophila* serogroup 1, strain Lp02 | [[6](#_ENREF_6)] |
| Lp02 “pentuple mutant” | Lp02 lacking gene clusters 3, 2ab, 6a, 7a, 4a | [[6](#_ENREF_6)] |
| *L. bozemanii* | Swiss National Reference Centre for *Legionella* (SNRCL) # 1165; Italy, 2002 (water) | [[7](#_ENREF_7)] |
| *L. longbeachae* | Strain NSW150 | [[8](#_ENREF_8)] |
| *L. micadei* | SNRCL #634; Bern, 2000 (patient) | This paper |
| *L. parisiensis* | Gift from Helge Bode | [[9](#_ENREF_9)] |
| *L. rubrilucens* | SNRCL #1318; Geneva, 2002 (water) | [[7](#_ENREF_7)] |
| *L. taurenesis* | SNRCL #1347; Bern, 2002 (water) | [[7](#_ENREF_7)] |
| *S. aureus* | ATCC25923 | ATCC |
| *V. cholerae* | Strain Vc2740-80, gift from Linda Thöny | [[10](#_ENREF_10)] |
| *S. flexneri* | Strain M90T | [[11](#_ENREF_11)] |
| *K. aerogenes* | Laboratory collection |  |
| *P. aeruginosa* | Strain PA01, gift from Alice Prince | [[12](#_ENREF_12)] |
| *S. typhimurum* | Strain C5 | [[13](#_ENREF_13)] |
| *Y. enterocolitica* | ATCC23715 | ATCC |
| *M. marinum* | *M. marinum* msp12::GFP, gift Lalita Ramakrishnan | [[14](#_ENREF_14)] |
| *M. tuberculosis* | H37Rv; ATCC 27294 | ATCC |

^a^ Abbreviations: Km, kanamycin resistance; Gm, gentamicin resistance.

**References**

1. Sadosky AB, Wiater LA, Shuman HA (1993) Identification of *Legionella pneumophila* genes required for growth within and killing of human macrophages. Infect Immun 61: 5361-5373.

2. Segal G, Shuman HA (1998) Intracellular multiplication and human macrophage killing by *Legionella pneumophila* are inhibited by conjugal components of IncQ plasmid RSF1010. Mol Microbiol 30: 197-208.

3. Li XH, Zeng YL, Gao Y, Zheng XC, Zhang QF, et al. (2010) The ClpP protease homologue is required for the transmission traits and cell division of the pathogen *Legionella pneumophila*. BMC Microbiol 10: 54.

4. Dietrich C, Heuner K, Brand BC, Hacker J, Steinert M (2001) Flagellum of *Legionella pneumophila* positively affects the early phase of infection of eukaryotic host cells. Infect Immun 69: 2116-2122.

5. Hales LM, Shuman HA (1999) The *Legionella pneumophila* *rpoS* gene is required for growth within *Acanthamoeba castellanii*. J Bacteriol 181: 4879-4889.

6. O'Connor TJ, Adepoju Y, Boyd D, Isberg RR (2011) Minimization of the *Legionella pneumophila* genome reveals chromosomal regions involved in host range expansion. Proc Natl Acad Sci U S A 108: 14733-14740.

7. Spirig T, Tiaden A, Kiefer P, Buchrieser C, Vorholt JA, et al. (2008) The *Legionella* autoinducer synthase LqsA produces an a-hydroxyketone signaling molecule. J Biol Chem 283: 18113-18123.

8. Cazalet C, Gomez-Valero L, Rusniok C, Lomma M, Dervins-Ravault D, et al. (2010) Analysis of the *Legionella longbeachae* genome and transcriptome uncovers unique strategies to cause Legionnaires' disease. PLoS Genetics 6: e1000851.

9. Ahrendt T, Miltenberger M, Haneburger I, Kirchner F, Kronenwerth M, et al. (2013) Biosynthesis of the natural fluorophore legioliulin from *Legionella*. Chembiochem. Epub ahead of print.

10. Almeida RJ, Cameron DN, Cook WL, Wachsmuth IK (1992) Vibriophage VcA-3 as an epidemic strain marker for the U.S. Gulf Coast *Vibrio cholerae* O1 clone. J Clin Microbiol 30: 300-304.

11. Sansonetti PJ, Kopecko DJ, Formal SB (1982) Involvement of a plasmid in the invasive ability of *Shigella flexneri*. Infect Immun 35: 852-860.

12. Cacalano G, Kays M, Saiman L, Prince A (1992) Production of the *Pseudomonas aeruginosa* neuraminidase is increased under hyperosmolar conditions and is regulated by genes involved in alginate expression. J Clin Invest 89: 1866-1874.

13. Hermant D, Ménard R, Arricau N, Parsot C, Popoff MY (1995) Functional conservation of the *Salmonella* and *Shigella* effectors of entry into epithelial cells. Mol Microbiol 17: 781-789.

14. Chan K, Knaak T, Satkamp L, Humbert O, Falkow S, et al. (2002) Complex pattern of *Mycobacterium marinum* gene expression during long-term granulomatous infection. Proc Natl Acad Sci U S A 99: 3920-3925.
